# Inequalities in somatic comorbidities among immigrants and Norwegians with and without common mental disorders: a national register study

**DOI:** 10.1007/s00127-025-02892-6

**Published:** 2025-04-03

**Authors:** Dawit Shawel Abebe, Kamila Angelika Hynek, Lars Lien, Anca Maria Yttri, Melanie Lindsay Straiton

**Affiliations:** 1 Research Centre for Substance Use Disorders and Mental Illness, Innlandet Hospital Trust, Brumunddal, Norway; 2 Department of Nursing and Health Promotion, Oslo Metropolitan University, Oslo, Norway; 3 Division for Mental and Physical Health, Norwegian Institute of Public Health, Postboks 222, Skøyen, Oslo 0213 Norway; 4 Faculty of Social and Health Sciences, Inland Norway University of Applied Sciences, Elverum, Norway

**Keywords:** Somatic disease, Mental disorder, Comorbidity, Immigrant, Registry, Norway

## Abstract

**Purpose:**

Comorbidity between mental disorders and somatic diseases exacerbates health outcomes and contributes to premature mortality. However, differences in this comorbidity among immigrant groups compared to the majority population are unclear. This study aims to examine disparities in the risk relationship between common mental disorders (CMDs) and somatic diseases among the majority population (Norwegians) and various immigrant groups.

**Methods:**

This national register study uses information from 3 142 925 residents aged 18+on diagnosed CMDs and selected somatic diseases for years 2008–2016. Poisson regression models were used to study the association between CMD and somatic diseases (i.e., cardiovascular diseases (CVDs), endocrine and metabolic diseases, cancer, and infectious diseases). Differences in risk between Norwegians and immigrant groups were investigated by introducing interaction terms between CMD and immigrant background.

**Results:**

Individuals with CMDs had a higher risk for all somatic diseases compared to those without, regardless of immigrant status. Immigrant groups varied in comorbidity, with those without CMDs showing similar or lower risk compared to Norwegians. However, immigrants with CMDs from non-Western countries (i.e., Eastern Europe, sub-Saharan Africa, South Asia) had a significantly higher probability of developing CVD, hypertension, and diabetes mellitus than Norwegians with CMDs. Additionally, SSA immigrants with CMDs also had a higher risk for viral hepatitis.

**Conclusion:**

Findings suggest that immigrant groups experience varying degrees of comorbidity, which underscores the need for tailored healthcare interventions to address these disparities effectively.

**Supplementary Information:**

The online version contains supplementary material available at 10.1007/s00127-025-02892-6.

## Introduction

Mental disorders are often associated with comorbid somatic conditions and diseases, both communicable and non-communicable [1, 2], with individuals with mental disorders being at increased risk of at least one somatic disorder requiring treatment [3]. Furthermore, individuals experiencing mental disorder are also more likely to engage in adverse health behaviours [4], and less likely to follow the prescribed treatment [5]. They, thus, have an increased risk of premature mortality, particularly due to somatic conditions [6] and between seven to twenty-four years of life lost when compared to individuals without mental disorder [7, 8]. However, the somatic health of individuals with mental disorders is often neglected [9] and undertreated [10].

Immigrants have increased risk of poor mental health [11–13], though there are differences within the immigrant population. For instance, refugees are at higher risk of common mental health disorders (CMDs) such as anxiety, depression and posttraumatic stress disorder (PTSD) than non-refugees [14]. Immigrants from Middle Eastern and former Yugoslavia countries have both a higher prevalence of self-reported and diagnosed mental disorders [15–17], whereas immigrants from Asia, Sub-Saharan Africa (SSA) and Eastern Europe seem to have lower prevalence [18–20]. There is also research reporting better mental but also somatic health of the immigrant population as compared to majority population in their new country of residency, commonly referred to as the *Healthy Immigrant Effect* (HIE) [21, 22]. Yet, a recent study from Sweden found this mental health advantage to only apply to immigrants from Western countries [17].

Regarding somatic diseases, research suggests that the burden is higher among some immigrant groups. For example, the prevalence of diabetes mellitus is found to be highest among South Asian immigrants and those from the Middle East and Africa [23, 24]. The risk of cardiovascular diseases (CVDs) is highest among South Asians and Eastern Europeans [24, 25], while overweight/obesity is most common among African and South Asian women, and Eastern European and Middle Eastern men [23, 24, 26]. Hypertension is most prevalent among SSA and South- and Southeast Asians [23, 24].

There is a large body of research investigating the association between mental disorders and in particular, CMDs, and a range of somatic conditions in the general population. CMDs include depression, generalised anxiety disorder, panic disorder, phobias, social anxiety disorder, obsessive-compulsive disorder, and PTSD [27]. A high prevalence of comorbidity was reported between depression and hypertension and metabolic disorders [28]. CMDs also increase the risk of endocrine, and nutritional diseases [28–30], a range of CVDs [28, 31, 32], cancer [3] and infectious diseases [33, 34]. Some of these comorbidities have been ascribed to common risk factors, such as genetic risk factors, limited access and use of healthcare services or poor adherence to medical treatments, stigma, and lifestyle factors (e.g., poor diet, substance abuse, and physical inactivity) that are more common among patients with mental disorders [35, 36]. These risk factors, e.g., limited access and use of healthcare services, poor lifestyle factors and stigma, are also particularly common among immigrants from countries outside of the European Economic Area, North America, Australia and New Zealand [37]. Thus, it would be reasonable to assume that some immigrant groups may be at particularly high risk of comorbidity between mental and somatic disorders.

Despite this, and the known excess risk of somatic comorbidities for individuals with CMDs, there is a lack of research investigating whether comorbidities between CMDs and somatic diseases differ across immigrant groups and the majority population. Only one recent study investigated the incidence of somatic diseases in several immigrant groups, with and without a diagnosis of PTSD and/or depression, and found that immigrants with PTSD/depression had higher rates of somatic disease, particularly infectious, neurological and pulmonary diseases [38]. However, this study only provided a within-group comparison and thus, we still lack knowledge about the differences between the majority population and immigrant groups.

By using national register data, this study aimed to investigate the burden of diagnosed somatic diseases among Norwegians and immigrants with CMDs as well as to examine differences between Norwegians and immigrant groups in the associations between diagnosed CMDs and somatic diseases. We included somatic diseases which have been identified as major comorbid somatic diseases in patients with CMDs, such as CVD, endocrine and metabolic disorders, cancer and infectious diseases [35, 39]. The investigated immigrant groups originate from European Economic Area, North America, Australia and New Zealand (Western countries), Eastern Europe, Middle East/North Africa (MENA), SSA, and South Asia.

## Methods

### Study design

The study is register-based (covering years 2008–2016), combining sociodemographic information from the National Population Registry and diagnosis information on somatic diseases and CMDs obtained from the Norwegian Patient Registry. Norwegian Patient Registry holds data on all registered diagnoses obtained during contacts with specialist health care services.

### Participants

The study participants consisted of Norwegians and immigrants aged 18 years or older who were legal residents in Norway on January 1, 2008 (*N* = 3 502 467). The Norwegian category refers to individuals born in Norway to two Norwegian-born parents (*N* = 2 768 685). Immigrants are defined as individuals born abroad to two foreign-born parents (*N* = 374 240). Those who were registered as deceased (*N* = 359 542) during the study period (2008–2016) were excluded from analysis. The final sample population consisted of 3 142 925 individuals.

### Outcomes

Diagnoses of somatic diseases were received during outpatient and inpatient contacts with specialist healthcare between 2008 and 2016. The diagnoses are based on the International Classification of Diseases, tenth revision (ICD-10). It included CVD, hypertension, diabetes, obesity, metabolic disorders, cancer, and infectious diseases, such as viral hepatitis, influenza and pneumonia and chronic lower respiratory diseases. Table 1 presents the dichotomous outcome variables representing ICD-10 diagnostic codes of somatic diseases.

**Table 1:** ICD-10 codes for outcome variables (somatic diseases)

Outcome variables	ICD-10 codes
Cardiovascular diseases	I10-I99
Hypertensive diseases	I10-I19
**Endocrine and metabolic diseases**	
Diabetes mellitus	E10-E14
Obesity	E66
Metabolic disorders	E70-E90
Cancer	C00-C96
**Infectious diseases**	
Viral Hepatitis	B15-B19
Influenza and pneumonia	J09-J18
Chronic lower respiratory diseases	J40-J47

ICD = International Classification of Diseases

### Explanatory variables

CMDs are diagnosed based on ICD-10, chapter V on Mental and behavioural disorders. An individual was coded as having a CMD if they have a diagnosis of depression (F320-F322 & F330-333), generalised anxiety disorder (F411), panic disorder (F410), phobias (F400-409), obsessive-compulsive disorder (F420-F429), or post-traumatic stress disorder (F431). Diagnoses were provided by psychologists or psychiatrists during outpatient and inpatient contacts with the specialist mental health care service between 2008 and 2016.

### Immigrant background

This variable consisted of Norwegians (individuals born in Norway to two Norwegian-born parents) and immigrants (individuals born abroad with two foreign-born parents) divided into five world region origins: Western countries (Sweden, Denmark, Finland, Island, Greenland/Faroe Islands, Austria, Belgium, Cyprus, France, Germany, Greece, Ireland, Italy, Luxemburg, Malta, Netherlands, Portugal, Spain, UK, Liechtenstein, Switzerland, Monaco, San Marino, Andorra, Guernsey, Isle of Man, Gibraltar, Vatican, Canada, United States, Australia, and New Zealand); Eastern Europe (Albania, Bulgaria, Eastland, Belarus, Croatia, Latavia, Poland, Romania, Lithuania, Moldova, Russia, Slovenia, Ukraine, Bosnia Herzegovina, Slovakia, Czech, Serbia, Montenegro, and Kosovo); MENA (Algeria, Bahrain, Egypt, Iran, Iraq, Israel, Jordan, Kuwait, Lebanon, Libya, Morocco, Palestine, Oman, Qatar, Saudi Arabia, Syria, Tunisia, Turkey, United Arab Emirates and Yemen); South Asia (Pakistan, India, Sri Lanka, Bangladesh, Afghanistan, Bhutan, Maldives, and Nepal), and SSA (Angola, Benin, Botswana, Burkina Faso, Burundi, Cape Verde, Cameroon, Central African Republic, Chad, Côte d’Ivoire, Democratic Republic of the Congo, Djibouti, Equatorial Guinea, Eritrea, Ethiopia, Gabon, Gambia, Ghana, Guinea, Guinea-Bissau, Kenya, Lesotho, Liberia, Madagascar, Malawi, Mali, Mauritania, Mauritius, Mozambique, Namibia, Niger, Nigeria, Republic of the Congo, Rwanda, Swaziland, São Tomé and Príncipe, Senegal, Seychelles, Sierra Leone, Somalia, South Africa, South Sudan, Tanzania, Togo, Uganda, Zambia, and Zimbabwe). The rationale for making this category is crucial for a detailed and nuanced understanding of inequalities in the burden of comorbidities in the immigrant population. The categorisation was based on a combination of geographical and cultural regions, but also with consideration to ensuring a sufficient number of migrants in each category. Migrants from other parts of the world were deemed as too diverse to be grouped together, or too small to be included.

### Covariates

Covariates include age (measured continuously), gender (sex at birth: males and females) and household poverty (an index for socioeconomic status (SES)). According to the EU-scale, household poverty is defined as less than 60% of the annual median disposable equivalised household income after adjustment for the number of household members [40]. A dummy variable (0 and 1) was constructed to indicate whether participants were defined as living in a household of poverty in each year. A sum score for all years was calculated with 0 being the lowest score (no years in poverty) and 9 being the highest sum score (in poverty all years in the study). A higher sum score indicates more years with household poverty between 2008 and 2016. This adjustment of household poverty accounts for the cumulative impact of socioeconomic disadvantage on comorbidity outcomes. Given the study’s focus on migrant background, it is particularly relevant, as the socio-economic trajectories across migrants and non-migrants may differ significantly.

### Data analysis

First, the characteristics of the study population by immigrant background, and the prevalence of both CMDs and specific somatic diseases were investigated (Table 2). Separate Poisson regression models were then applied to estimate the incidence rate ratios (IRR) of the association between CMD and somatic diseases. We then conducted stepwise modelling where we calculated the risk of each somatic disease by CMD status (Model 1), by immigrant background (Model 2), and an interaction term between CMD status and immigrant background in the Model 3. The interaction term was applied to investigate the differences in the risk of somatic diseases among individuals with and without a CMD by immigrant background. Regression estimates (IRR with 95% confidence intervals (CI)) were adjusted for age, gender, and household poverty, with Norwegians as the reference group (Table 3). The interaction results are presented as marginal probabilities in Table 4. The analyses were performed by means of Stata SE/16.

**Table 2:** Characteristics of the study population, by region of origin

	Total *N* (%)	Norwegian *N* (%)	Western *N* (%)	Eastern Europe *N* (%)	Middle East/ North Africa *N* (%)	Sub-Saharan Africa *N* (%)	South Asia *N* (%)
N (%)	3 197 182	2 768 685 (86.6)	144 019 (4.9)	158 487 (4.9)	48 514 (1.5)	38 228 (1.2)	38 228 (1.2)
Male	1 558 504 (49.6)	1 365 480 (49.3)	79 248 (55.0)	101 376 (63.9)	29 399 (60.6)	22 567 (57.5)	21 828 (57.1)
Female	1 584 421 (50.4)	1 403 205 (50.7)	64 771 (45.0)	57 111 (36.1)	19 115 (39.4)	16 682 (42.5)	16 400 (42.9)
Age, Mean (SD)	46.5 (16.4)	46.6 (16.4)	39.8 (15.1)	34.1 (11.2)	36.9 (11.5)	33.4 (10.7)	38.0 (12.7)
Household poverty index, Mean (SD)	0.5 (1.3)	0.4 (1.2)	0.2 (1.0)	0.3 (1.2)	1.2 (2.1)	1.1 (2.1)	0.8 (1.8)
Common mental disorders	202 682 (6.4)	181 013 (6.5)	6123 (4.2)	5285 (3.3)	5908 (12.2)	1292 (3.3)	2162 (5.6)
Cardiovascular diseases	798 857 (25.4)	746 617 (26.9)	21 290 (14.8)	13 834 (8.7)	7202 (14.8)	3864 (9.8)	6280 (16.4)
Hypertensive diseases	372 579 (11.9)	351 053 (12.7)	9222 (6.4)	5118 (3.2)	2331 (4.9)	1567 (3.9)	3086 (8.1)
**Endocrine and metabolic disorders**							
Diabetes mellitus	173 423 (5.5)	155 237 (5.6)	4101 (2.8)	2813 (1.8)	3039 (6.3)	1980 (5.0)	4638 (12.1)
Obesity	68 583 (2.2)	63 577 (2.3)	1605 (1.1)	1138 (0.7)	1061 (2.2)	486 (1.2)	720 (1.8)
Metabolic disorders	203 629 (6.5)	191 277 (6.9)	4966 (3.4)	2649 (1.7)	1781 (3.7)	952 (2.4)	1761 (4.6)
Cancer	264 012 (8.4)	252 154 (9.1)	6977 (4.8)	2587 (1.6)	993 (2.1)	530 (1.4)	741 (1.9)
**Infectious diseases**							
Viral Hepatitis	15 976 (0.5)	11 463 (0.4)	350 (0.2)	1004 (0.6)	518 (1.1)	1221 (3.1)	505 (1.3)
Influenza and pneumonia	110 975 (3.5)	103 733 (3.8)	2686 (1.9)	1448 (0.9)	1089 (2.2)	853 (2.2)	1080 (2.8)
Chronic lower respiratory diseases	177 476 (5.7)	166 333 (6.0)	4383 (3.0)	1859 (1.2)	1794 (3.7)	808 (2.1)	1683 (4.4)

SD– standard deviation; N– number

**Table 3:** Incidence risk ratio (IRR) with 95% confidence intervals (95% CI) of somatic diseases. Adjusted for age, gender and household poverty

	Cardiovascular diseases		Hypertensive diseases		Diabetes mellitus		Obesity		Metabolic disorders		Cancer		Viral hepatitis		Influenza and pneumonia		Chronic lower respiratory diseases	
	IRR	95% CI	IRR	95% CI	IRR	95% CI	IRR	95% CI	IRR	95% CI	IRR	95% CI	IRR	95% CI	IRR	95% CI	IRR	95% CI
**Model 1**																		
CMD (ref.no)	1.4	1.39–1.42	1.4	1.36–1.40	1.45	1.42–1.47	2.43	2.38–2.48	2.02	1.99–2.04	1.15	1.13–1.17	3.03	2.91–3.16	2.04	1.99–2.08	1.83	1.80–1.84
**Model 2**																		
**Immigrant background**																		
Norwegians (ref.)	1		1		1		1		1		1		1		1		1	
Western	0.73	0.72–0.74	0.74	0.73–0.76	0.67	0.65–0.69	0.47	0.45–0.50	0.67	0.65–0.69	0.76	0.74–0.78	0.61	0.54–0.67	0.69	0.66–0.72	0.64	0.62–0.66
Eastern Europe	0.61	0.59–0.62	0.63	0.61–0.65	0.59	0.57–0.61	0.31	0.29–0.33	0.47	0.45–0.49	0.41	0.40–0.43	1.42	1.33–1.52	0.52	0.49–0.54	0.32	0.31–0.34
Middle East/North Africa	0.92	0.89–0.94	0.82	0.78–0.85	1.79	1.73–1.85	0.91	0.85–0.96	0.84	0.81–0.88	0.48	0.45–0.51	1.58	1.45–1.74	0.97	0.92–1.04	0.87	0.83–0.91
Sub-Saharan Africa	0.74	0.71–0.76	0.88	0.84–0.93	1.81	1.72–1.87	0.53	0.49–0.58	0.71	0.67–0.76	0.39	0.36–0.42	5.51	5.18–5.86	1.26	1.18–1.35	0.58	0.54–0.62
South Asia	0.95	0.93–0.97	1.23	1.19–1.27	3.34	3.25–3.43	0.81	0.76–0.88	1.05	0.98–1.09	0.40	0.37–0.43	2.58	2.36–2.83	1.22	1.15–1.29	1.02	0.98–1.07

IRR– Incidence risk ratio; 95% CI– 95% confidence interval; CMD– Common mental disorders

**Table 4:** Predictive margins based on the interaction analysis for CMD and somatic diseases. Adjusted for age, gender and household poverty

	Norwegian		Western		Eastern Europe		Middle East/ North Africa		Sub-Saharan Africa		South Asia	
	Pr	95% CI	Pr	95% CI	Pr	95% CI	Pr	95% CI	Pr	95% CI	Pr	95% CI
**Cardiovascular diseases**												
No CMD	0.25	0.25–0.25	0.18	0.18–0.19	0.14	0.14–0.15	0.22	0.22–0.23	0.18	0.18 − 0.17	0.23	0.23–0.24
CMD	0.34	0.34–0.35	0.29	0.28–0.30	0.40	0.38–0.42	0.34	0.32–0.36	0.33	0.29–0.37	0.41	0.38–0.44
**Hypertensive diseases**												
No CMD	0.12	0.12–0.12	0.08	0.08–0.09	0.07	0.06–0.07	0.10	0.09–0.10	0.10	0.09–0.11	0.14	0.14–0.15
CMD	0.16	0.15–0.16	0.13	0.12–0.14	0.18	0.17–0.20	0.12	0.11–0.14	0.18	0.14–0.21	0.25	0.21–0.27
**Diabetes mellitus**												
No CMD	0.05	0.05–0.05	0.03	0.03–0.04	0.03	0.03–0.03	0.10	0.09–0.10	0.09	0.09–0.10	0.17	0.16–0.17
CMD	0.07	0.07–0.07	0.06	0.05–0.06	0.09	0.08–0.10	0.12	0.11–0.13	0.17	0.13–0.19	0.25	0.23–0.27
**Obesity**												
No CMD	0.02	0.02–0.02	0.01	0.01–0.01	0.01	0.01–0.01	0.02	0.02–0.02	0.01	0.01–0.01	0.02	0.01-0.0
CMD	0.05	0.04–0.05	0.03	0.03–0.04	0.03	0.02–0.03	0.03	0.03–0.04	0.03	0.02–0.04	0.04	0.03–0.05
**Metabolic disorders**												
No CMD	0.06	0.06–0.06	0.04	0.04–0.04	0.03	0.03–0.03	0.05	0.05–0.06	0.04	0.04–0.05	0.06	0.06–0.07
CMD	0.12	0.12–0.13	0.09	0.08–0.10	0.10	0.09–0.11	0.10	0.09–0.11	0.12	0.09–0.14	0.13	0.12–0.15
**Cancer**												
No CMD	0.08	0.08–0.09	0.06	0.06–0.07	0.03	0.03–0.03	0.04	0.03–0.04	0.03	0.03–0.03	0.03	0.03–0.04
CMD	0.09	0.09–0.10	0.09	0.08–0.09	0.07	0.06–0.08	0.06	0.05–0.07	0.06	0.04–0.08	0.05	0.04–0.07
**Vital Hepatitis**												
No CMD	< 0.01	< 0.01–0.01	< 0.01	< 0.01–0.01	0.01	0.01–0.01	0.01	< 0.01–0.01	0.02	0.02–0.02	0.01	0.01–0.01
CMD	0.01	0.01–0.01	0.01	0.01–0.01	0.02	0.01–0.02	0.01	0.01–0.01	0.03	0.03–0.04	0.02	0.01–0.03
**Influenza and pneumonia**												
No CMD	0.08	0.08–0.09	0.06	0.06–0.07	0.03	0.03–0.03	0.04	0.03–0.04	0.03	0.03–0.03	0.03	0.03–0.04
CMD	0.09	0.09–0.10	0.09	0.08–0.09	0.07	0.06–0.08	0.06	0.05–0.07	0.06	0.04–0.08	0.05	0.04–0.07
**Chronic lower respiratory diseases**												
No CMD	0.08	0.08–0.09	0.06	0.06–0.07	0.03	0.03–0.03	0.04	0.03–0.04	0.03	0.03–0.03	0.03	0.03–0.04
CMD	0.09	0.09–0.10	0.09	0.08–0.09	0.07	0.06–0.08	0.06	0.05–0.07	0.06	0.04–0.08	0.05	0.04–0.07

Pr = Predictive probability; 95% CI– 95% confidence interval; CMD– Common mental disorders

## Results

### Characteristics of the study population

The descriptive summary of the study population, by immigrant background, is presented in Table 2. From January 1, 2008, to December 31, 2016, 6.4% (*N* = 202 682) of the study population had a CMD diagnosis. The highest percentage with a diagnosed CMD originated from MENA (12.2%) and lowest percentage from Eastern Europe and SSA (3.3%). For the most studied somatic diseases, the general pattern was that immigrants had a lower prevalence than, or comparable prevalence to the Norwegians. There were, however, some marked exceptions. Diabetes mellitus was more prevalent among South Asian immigrants compared to Norwegians (12.1% vs. 5.6%). Further, viral hepatitis was more common among SSA immigrants than Norwegians (3.1% vs. 0.4%).

### Differences in the risk of somatic diseases between Norwegians and immigrants

Table 3 presents the results of Poisson regression models for the IRR (95% CI) for each somatic disease, categorized by CMD status and immigrant background, with Norwegians as the reference group. Estimates were adjusted for age, gender, and household poverty. Model 1 revealed that individuals with a CMD had a significantly higher risk for all somatic diseases compared to those without a CMD. In Model 2, immigrants from Western and Eastern European countries showed lower or comparable risk for all investigated somatic diseases compared to Norwegians, except for viral hepatitis, which was significantly higher among Eastern European immigrants. Immigrants from MENA, SSA, and South Asia exhibited significantly higher risks of diabetes mellitus and viral hepatitis. Moreover, immigrants from SSA and South Asia also had a significantly higher risk of influenza and pneumonia. Notably, this risk of cancer was particularly low for immigrants from MENA, SSA and South Asia compared with Norwegians.

To investigate the difference in the risk of comorbidity between CMDs and somatic diseases by immigrant background, an interaction term between CMD status and immigrant background was added to Model 3. We found significant interactions between CMD status and immigrant background for many somatic diseases (see supplementary Table 1). Interaction terms are presented as marginal probabilities in Table 4 illustrating the marginal probabilities of somatic diseases among Norwegians and immigrants with and without CMDs. These interaction findings are also presented in the forest plots for those with and without CMDs separately (see supplementary Figs. 1 and 2).

The probability of most somatic diseases was significantly higher among individuals with a CMD, regardless of immigrant status. Some immigrant groups with a CMD demonstrated a notably higher probability of CVD, hypertension, and diabetes mellitus. Specifically, South Asian and Eastern European immigrants with a CMD had significantly higher rates of CVD, while Eastern European, SSA, and South Asian immigrants with a CMD exhibited significantly higher rates of hypertension. Additionally, Eastern European, MENA, SSA, and South Asian immigrants with a CMD had significantly higher rates of diabetes. Moreover, SSA immigrants with a CMD showed a significantly higher probability of viral hepatitis. The majority of differences in the probability of these somatic diseases between Norwegians and immigrant groups without a CMD were either non-significant or lower.

## Discussion

In this study, we examined disparities in the risk of somatic diseases based on immigrant background and the comorbidity between CMDs and somatic diseases diagnosed in specialist healthcare between 2008 and 2016 in Norway. Immigrants displayed either similar or significantly lower risk compared to Norwegians for the majority of examined somatic diseases. However, immigrants from MENA, SSA, and South Asia exhibited an elevated risk of diabetes mellitus and infectious diseases (viral hepatitis or influenza and pneumonia). This higher risk among these immigrants for some of these diseases has been documented in previous research (e.g., 23, 24, 25).

Particular interest was paid to potential disparities in comorbidity between Norwegians and different immigrant groups. Through interaction analyses and results presented as predictive margins, we observed significant differences in the likelihood of CVD, hypertension, diabetes mellitus, and viral hepatitis based on CMD status and immigrant background. Relative to Norwegians, the disparities in the likelihood of CVD, hypertension, or diabetes mellitus were more pronounced among Eastern European, SSA, and South Asian immigrants with a CMD than among those without.

Potential reasons for the increased risk of these somatic diseases among immigrants from non-Western countries with CMDs include poor lifestyle choices, such as unhealthy dietary habits and physical inactivity [37] and low health literacy, which may be more prevalent among these immigrants compared to other immigrant groups [41, 42]. Individuals with a CMD may also encounter greater challenges in navigating the healthcare system, leading to delayed access to services and potentially exacerbated health outcomes upon seeking care. Norway has a universal healthcare system that covers both primary and specialist healthcare services. However, user fees (*egenandel*) are required for consultations, diagnostic tests, and certain treatments until an annual cost ceiling (*frikort*) is reached, after which services become free for the rest of the year. While this system ensures affordability for most residents, immigrants may face challenges such as limited awareness of their healthcare rights, administrative hurdles, and financial constraints before qualifying for exemption. These factors can contribute to disparities in healthcare access and utilization among immigrant populations. Additionally, immigrants from non-Western countries have poorer living conditions than the general population [43], and the acculturation stress [44] and stigma [45] associated with mental disorders may be particularly pronounced, resulting in heightened risk for comorbidities and illness severity.

The relative differences in risk of somatic diseases between Norwegians and most immigrant groups without a CMD were, however, lower. This finding is in accordance with other studies suggesting that immigrants have lower odds of multimorbidity than non-immigrant populations [46, 47]. Alternatively, the general lower risk of somatic diseases among immigrants without a CMD may be a result of screening practices among some immigrant groups (e.g., cancer), poorer help-seeking behaviours for both mental and somatic diseases, help-seeking at later stages [48–51], or better health compared to the Norwegians (i.e. HIE) [52]. Moreover, immigrants are less likely than Norwegians to use specialist healthcare help for mental disorders [53–55]. It’s imperative to recognize that out-of-pocket payment for health services may serve as a barrier for certain immigrants with CMDs. Thus, there may be more undiagnosed CMDs among immigrants than Norwegians, which could underestimate some of the inequalities in the risk of somatic diseases among those without a CMD.

### Limitations

First, the methodology applied to study the association between CMDs, and the risk of somatic comorbidity does not allow us to determine the causal path. As both CMDs and somatic disorders could be diagnosed at any time of the study follow-up, we cannot say which was the first. We did not consider whether individuals had multiple mental disorders and/or multiple somatic diseases either. Second, the dataset did not allow identification of individuals who emigrated during the follow-up period. If many immigrants migrate home when they experience serious illness (mental or somatic), as suggested by the Salmon bias hypothesis [56], we may be somewhat underestimating the risk of CMDs and somatic disorders as well as the level of comorbidities. However, this would only have a limited impact on the results, particularly for non-Western immigrants as the out-migration for this group is very low [57]. Third, as reported in previous research, immigrants may be at increased risk of some but not necessarily all types of CMD. Thus, focus on more specific diagnosis could provide an even more nuanced result. However, the use of specific CMD as well as a range of somatic diseases could threaten the statistical power of the analyses. Furthermore, it is important to reflect that underdiagnoses of some diseases may be present. This may particularly apply to CMDs among immigrants, who appear less likely to use mental healthcare [53, 58, 59]. Lastly, we recognize that significant diversity within the five broad world regions may influence our results, potentially masking important differences among migrants. As a result, this categorization is not optimal. The within-group variations may be substantial, and a more detailed classification—such as specific measures of ethnicity or country of origin—is needed to better understand how and why these groups may differ.

This study, however, has several strengths, such as the methodological advantages that the use of Norwegian Patient Registry brings. First, the coverage of healthcare services and the quality of health records in Norway is considered to be high, which facilitates representativeness and reduces selection bias [60]. Second, a larger study population brings adequate statistical power to detect the heterogeneity in the clinical epidemiology of some relatively rare somatic diseases across several immigrant groups with and without mental disorders. Further, all diagnoses were based on clinically set diagnosis from specialist care, and not self-reported information thus reducing the risk of recall bias.

## Conclusions

In conclusion, this study on the comorbidity between CMDs and various somatic disorders among immigrants and Norwegians is of great importance as it highlights which groups may be at greatest risk of comorbid disorders and related consequences. Although the differences in risk of comorbidity between mental and somatic diseases were, to a large extent, non-significant or slightly lower among immigrants than Norwegians, there are groups that show higher risk. Further, the largest relative differences between Norwegians and the studied immigrant groups were found among those with a CMD. This was highly marked for immigrants from non-Western countries. Thus, the healthcare system should give greater attention to individuals with CMD. In particular, targeted interventions addressing early screening among psychiatric patients in groups that have a higher risk of somatic diseases should be given a priority. An additional crucial clinical implication is the necessity to ensure access to regular examinations by general practitioners for individuals at elevated risk of somatic disorders. Future research should aim to better understand the mechanisms behind these inequalities in order to find out if the differences in mental and somatic comorbidities may be a result of specific risk factors, barriers regarding healthcare help-seeking among immigrants, underdiagnoses or better health of immigrants than of Norwegians.

## Electronic supplementary material

Below is the link to the electronic supplementary material.

Supplementary Material 1

## Data Availability

No datasets were generated or analysed during the current study.

## References

[1] Allen J, Balfour R, Bell R, Marmot M (2014) Social determinants of mental health. Int Rev Psychiatry 26(4):392–407 25137105 10.3109/09540261.2014.928270

[2] Prince M, Patel V, Saxena S, Maj M, Maselko J, Phillips MR et al (2007) No health without mental health. Lancet 370(9590):859–877 17804063 10.1016/S0140-6736(07)61238-0

[3] Lawrence D, Hancock KJ, Kisely S (2013) The gap in life expectancy from preventable physical illness in psychiatric patients in Western Australia: retrospective analysis of population based registers. BMJ 346:f2539 23694688 10.1136/bmj.f2539 PMC3660620

[4] Velten J, Lavallee KL, Scholten S, Meyer AH, Zhang X-C, Schneider S et al (2014) Lifestyle choices and mental health: a representative population survey. BMC Psychol 2(1):58 25628891 10.1186/s40359-014-0055-y PMC4304169

[5] DiMatteo MR, Lepper HS, Croghan TW (2000) Depression is a risk factor for noncompliance with medical treatment: meta-analysis of the effects of anxiety and depression on patient adherence. Arch Intern Med 160(14):2101–2107 10904452 10.1001/archinte.160.14.2101

[6] Lien L, Huus G, Morken G (2015) Psykisk Syke lever kortere [Mentally ill live shorter]. Tidsskrift Den Norske Legeforening 135(3):246–248 10.4045/tidsskr.14.0831 25668543

[7] Chesney E, Goodwin GM, Fazel S (2014) Risks of all-cause and suicide mortality in mental disorders: a meta-review. World Psychiatry 13(2):153–160 24890068 10.1002/wps.20128 PMC4102288

[8] Ringen PA, Engh JA, Birkenaes AB, Dieset I, Andreassen OA (2014) Increased mortality in schizophrenia due to cardiovascular Disease– A Non-Systematic review of epidemiology, possible causes, and interventions. Front Psychiatry.;5 10.3389/fpsyt.2014.00137 PMC4175996 25309466

[9] Fleischhacker WW, Cetkovich-Bakmas M, De Hert M, Hennekens CH, Lambert M, Leucht S et al (2008) Comorbid somatic illnesses in patients with severe mental disorders: clinical, policy, and research challenges. J Clin Psychiatry 69(4):514–519 18370570 10.4088/jcp.v69n0401

[10] Mitchell AJ, Lord O, Malone D (2012) Differences in the prescribing of medication for physical disorders in individuals with V. without mental illness: meta-analysis. Br J Psychiatry 201(6):435–443 23209089 10.1192/bjp.bp.111.094532

[11] Close C, Kouvonen A, Bosqui T, Patel K, O’Reilly D, Donnelly M (2016) The mental health and wellbeing of first generation migrants: a systematic-narrative review of reviews. Globalization Health 12(1):47 27558472 10.1186/s12992-016-0187-3 PMC4997738

[12] Kjøllesdal M, Straiton ML, Øien-Ødegaard C, Aambø A, Holmboe O, Johansen R et al (2019) [Health among immigrants in Norway] Oslo: Folkehelseinstituttet

[13] Evans CR, Erickson N (2019) Intersectionality and depression in adolescence and early adulthood: A MAIHDA analysis of the National longitudinal study of adolescent to adult health, 1995–2008. Soc Sci Med 220:1–11 30390469 10.1016/j.socscimed.2018.10.019

[14] Henkelmann J-R, de Best S, Deckers C, Jensen K, Shahab M, Elzinga B et al (2019) Mental disorders in refugees. A Systematic Review and Meta-Analysis 10.1192/bjo.2020.54 PMC7443922 32611475

[15] Toselli S, Gualdi-Russo E, Marzouk D, Sundquist J, Sundquist K (2014) Psychosocial health among immigrants in central and Southern Europe. Eur J Pub Health 24(suppl1):26–30 10.1093/eurpub/cku100 25107995

[16] Abebe DS, Lien L, Hjelde KH (2014) What we know and don’t know about mental health problems among immigrants in Norway. J Immigr Minor Health 16(1):60–67 23117694 10.1007/s10903-012-9745-9

[17] Helgesson M, Johansson B, Nordquist T, Vingård E, Svartengren M (2019) Healthy migrant effect in the Swedish context: a register-based, longitudinal cohort study. BMJ Open 9(3):e026972 30878993 10.1136/bmjopen-2018-026972 PMC6429895

[18] Ekeberg KA, Abebe DS (2020) Mental disorders among young adults of immigrant background: a nationwide register study in Norway. Social Psychiatry and Psychiatric Epidemiology 10.1007/s00127-020-01980-z PMC8192316 33156357

[19] Markkula N, Lehti V, Gissler M, Suvisaari J (2017) Incidence and prevalence of mental disorders among immigrants and native Finns: a register-based study. Soc Psychiatry Psychiatr Epidemiol 52(12):1523–1540 28856385 10.1007/s00127-017-1432-7

[20] Hollander A-C, Bruce D, Burström B, Ekblad S (2013) The association between immigrant subgroup and poor mental health: a population-based register study. J Nerv Ment Dis 201(8):645–652 23896844 10.1097/NMD.0b013e31829dbd64

[21] Salas-Wright CP, Vaughn MG, Goings TC, Miller DP, Schwartz SJ (2018) Immigrants and mental disorders in the united States: new evidence on the healthy migrant hypothesis. Psychiatry Res 267:438–445 29980122 10.1016/j.psychres.2018.06.039 PMC6131041

[22] Dhadda A, Greene G (2018) The healthy migrant effect’ for mental health in England: Propensity-score matched analysis using the EMPIRIC survey. J Immigr Minor Health 20(4):799–808 28389831 10.1007/s10903-017-0570-z PMC6061089

[23] Cainzos-Achirica M, Vela E, Cleries M, Bilal U, Mauri J, Pueyo MJ et al (2019) Cardiovascular risk factors and disease among non-European immigrants living in Catalonia. Heart 105(15):1168 30819763 10.1136/heartjnl-2018-314436

[24] Commodore-Mensah Y, Selvin E, Aboagye J, Turkson-Ocran R-A, Li X, Himmelfarb CD et al (2018) Hypertension, overweight/obesity, and diabetes among immigrants in the united States: an analysis of the 2010–2016 National health interview survey. BMC Public Health 18(1):773 29925352 10.1186/s12889-018-5683-3 PMC6011357

[25] Rabanal KS, Lindman AS, Selmer RM, Aamodt G (2013) Ethnic differences in risk factors and total risk of cardiovascular disease based on the Norwegian CONOR study. Eur J Prev Cardiol 20(6):1013–1021 22642981 10.1177/2047487312450539

[26] Qureshi SA, Straiton ML, Gele AA (2020) Associations of socio-demographic factors with adiposity among immigrants in Norway: a secondary data analysis. BMC Public Health 20(1):772 32448125 10.1186/s12889-020-08918-9 PMC7247236

[27] National Collaborating Centre for Mental Health (UK) (2011) Common mental health disorders: identification and pathways to care. Common mental health disorders: identification and pathways to care. National Institute for health and care.Excellence: guidelines. British Psychological Society, Leicester (UK) 22536621

[28] Steffen A, Nübel J, Jacobi F, Bätzing J, Holstiege J (2020) Mental and somatic comorbidity of depression: a comprehensive cross-sectional analysis of 202 diagnosis groups using German nationwide ambulatory claims data. BMC Psychiatry 20(1):142 32228541 10.1186/s12888-020-02546-8 PMC7106695

[29] Dedert EA, Calhoun PS, Watkins LL, Sherwood A, Beckham JC (2010) Posttraumatic stress disorder, cardiovascular, and metabolic disease: A review of the evidence. Ann Behav Med 39(1):61–78 20174903 10.1007/s12160-010-9165-9 PMC3219414

[30] Eaton WW, Armenian H, Gallo J, Pratt L, Ford DE (1996) Depression and risk for onset of type II diabetes: A prospective population-based study. Diabetes Care 19(10):1097 8886555 10.2337/diacare.19.10.1097

[31] Goodman E, Whitaker RC (2002) A prospective study of the role of depression in the development and persistence of adolescent obesity. Pediatrics 110(3):497 12205250 10.1542/peds.110.3.497

[32] Theodosis-Nobelos P, Asimakopoulou E, Madianos M (2021) [Pathophysiological mechanisms of major mental disorders related to cardiovascular disease]. Psychiatriki 10.22365/jpsych.2021.038 34390565

[33] Cohen S, Williamson GM (1991) Stress and infectious disease in humans. Psychol Bull 109(1):5–24 2006229 10.1037/0033-2909.109.1.5

[34] Witthauer C, Gloster AT, Meyer AH, Goodwin RD, Lieb R (2014) Comorbidity of infectious diseases and anxiety disorders in adults and its association with quality of life: a community study. Front Public Health 2:104320 10.3389/fpubh.2014.00080 PMC4095564 25072049

[35] Firth J, Siddiqi N, Koyanagi A, Siskind D, Rosenbaum S, Galletly C et al (2019) The lancet psychiatry commission: a blueprint for protecting physical health in people with mental illness. Lancet Psychiatry 6(8):675–712 31324560 10.1016/S2215-0366(19)30132-4

[36] O’Neil A, Jacka FN, Quirk SE, Cocker F, Taylor CB, Oldenburg B et al (2015) A shared framework for the common mental disorders and non-communicable disease: key considerations for disease prevention and control. BMC Psychiatry 15:1–6 25652365 10.1186/s12888-015-0394-0 PMC4342822

[37] Abebe DS (2010) Public health challenges of immigrants in Norway: a research review. NAKMI Rep 2:2010

[38] Lolk M, Byberg S, Carlsson J, Norredam M (2016) Somatic comorbidity among migrants with posttraumatic stress disorder and depression– a prospective cohort study. BMC Psychiatry 16(1):447 27964720 10.1186/s12888-016-1149-2 PMC5153678

[39] Hert M, Correll CU, Bobes J, Cetkovich-Bakmas M, Cohen D, Asai I et al (2011) Physical illness in patients with severe mental disorders. I. Prevalence, impact of medications and disparities in health care. World Psychiatry 10(1):52–77 21379357 10.1002/j.2051-5545.2011.tb00014.x PMC3048500

[40] Eurostat, Glossary At-risk-of-poverty rate 2018 2018 [Available from: https://ec.europa.eu/eurostat/statistics-explained/index.php?title=Glossary:At-risk-of-poverty_rate#:~:text=The%20at%2Drisk%2Dof%2D,disposable%20income%20after%20social%20transfers

[41] Ramos NNV, Kielmann K, Martins MRO, Fronteira I (2023) Health literacy in African countries: a scoping review

[42] Le C, Finbråten HS, Pettersen KS, Joranger P, Guttersrud Ø (2021) Health Literacy in Five Immigrant Populations in Norway: Pakistan, Poland, Somalia, Turkey, and Vietnam. English summary. Helsekompetansen i fem innvandrerpopulasjoner i Norge Pakistan, Polen, Somalia, Tyrkia og Vietnam: Befolkningens helsekompetanse, del II

[43] Vrålstad S, Wiggen KS (2017) [Living conditions among persons with an immigrant background 2016]. Statistics Norway, Oslo, Norway, p 8253795394. Report No

[44] Choy B, Arunachalam K, Gupta S, Taylor M, Lee A (2021) Systematic review: acculturation strategies and their impact on the mental health of migrant populations. Public Health Pract 2:100069 10.1016/j.puhip.2020.100069 PMC9461568 36101596

[45] Eylem O, De Wit L, Van Straten A, Steubl L, Melissourgaki Z, Danışman GT et al (2020) Stigma for common mental disorders in Racial minorities and majorities a systematic review and meta-analysis. BMC Public Health 20:1–20 32513215 10.1186/s12889-020-08964-3 PMC7278062

[46] Diaz E, Poblador-Pou B, Gimeno-Feliu L-A, Calderón-Larrañaga A, Kumar BN, Prados-Torres A (2015) Multimorbidity and its patterns according to immigrant origin. A nationwide Register-Based study in Norway. PLoS ONE 10(12):e0145233 26684188 10.1371/journal.pone.0145233 PMC4684298

[47] Lenzi J, Avaldi VM, Rucci P, Pieri G, Fantini MP (2016) Burden of Multimorbidity in relation to age, gender and immigrant status: a cross-sectional study based on administrative data. BMJ Open 6(12):e012812 28003289 10.1136/bmjopen-2016-012812 PMC5223687

[48] Lagerlund M, Maxwell AE, Bastani R, Thurfjell E, Ekbom A, Lambe M (2002) Sociodemographic predictors of non-attendance at invitational mammography screening– a population-based register study (Sweden). Cancer Causes Control 13(1):73–82 11899121 10.1023/a:1013978421073

[49] Lee HY, Lundquist M, Ju E, Luo X, Townsend A (2011) Colorectal cancer screening disparities in Asian Americans and Pacific Islanders: which groups are most vulnerable? Ethn Health 16(6):501–518 22050536 10.1080/13557858.2011.575219

[50] Crawford J, Ahmad F, Beaton D, Bierman AS (2016) Cancer screening behaviours among South Asian immigrants in the UK, US and Canada: a scoping study. Health Soc Care Community 24(2):123–153 25721339 10.1111/hsc.12208

[51] Davis LE, Bogner E, Coburn NG, Hanna TP, Kurdyak P, Groome PA et al (2020) Stage at diagnosis and survival in patients with cancer and a pre-existing mental illness: a meta-analysis. J Epidemiol Community Health 74(1):84 31653661 10.1136/jech-2019-212311

[52] Kennedy S, Kidd MP, McDonald JT, Biddle N (2015) The healthy immigrant effect: patterns and evidence from four countries. J Int Migration Integr 16(2):317–332

[53] Abebe DS, Lien L, Elstad JI (2017) Immigrants’ utilization of specialist mental healthcare according to age, country of origin, and migration history: a nation-wide register study in Norway. Soc Psychiatry Psychiatr Epidemiol 52(6):679–687 28378064 10.1007/s00127-017-1381-1

[54] Elstad JI, Finnvold JE, Texmon I (2015) [Use of hospitals and other specialized medical services among immigrants and non-immigrants in Norway]. NOVA, OsloMet., Oslo

[55] Castaneda AE, Çilenti K, Rask S, Lilja E, Skogberg N, Kuusio H et al (2020) Migrants are underrepresented in mental health and rehabilitation Services-Survey and Register-Based findings of Russian, Somali, and Kurdish origin adults in Finland. Int J Environ Res Public Health.;17(17) 10.3390/ijerph17176223 PMC7504052 32867157

[56] Lu Y, Qin L (2014) Healthy migrant and salmon bias hypotheses: A study of health and internal migration in China. Soc Sci Med 102:41–48 24565140 10.1016/j.socscimed.2013.11.040

[57] Statistics Norway Migrations 2020 [Available from: https://www.ssb.no/en/befolkning/statistikker/flytting

[58] Patel K, Bosqui T, Kouvonen A, Donnelly M, Väänänen A, Bell J et al (2020) Unmet need for mental health medication within the migrant population of Northern Ireland: a record linkage study. J Epidemiol Community Health.:jech–2019 10.1136/jech-2019-212774 33130576

[59] Straiton ML, Corbett K, Hollander A-C, Hauge LJ (2019) Outpatient mental healthcare service use among women with migrant background in Norway: a National register study. BMC Health Serv Res 19(1):944 31818291 10.1186/s12913-019-4788-4 PMC6902575

[60] Thygesen L, Ersbøll A (2014) When the entire population is the sample: strengths and limitations in register-based epidemiology. Affiliated Eur Epidemiol Federation 29(8):551–558 10.1007/s10654-013-9873-0 24407880

