# Supplementary material for: Inequalities in somatic comorbidities among immigrants and Norwegians with and without common mental disorders: a national register study
Source: Soc Psychiatry Psychiatr Epidemiol. 2025 Apr 3;60(11):2653–62. doi: 10.1007/s00127-025-02892-6 (PMC12572047; doi:10.1007/s00127-025-02892-6)
Supplement: Supplementary file 1 — Supplementary Material 1 [file 127_2025_2892_MOESM1_ESM.docx]

**Supplmentary** **Table S1.** Incidence risk ratio (IRR) with 95% confidence intervals (95% CI) of somatic diseases based on the interaction between CMD status and immigrant background. Adjusted for age, gender and household poverty.

|  | **Cardiovascular diseases** | | **Hypertensive diseases** | | **Diabetes mellitus** | | **Obesity** | | **Metabolic disorders** | | **Cancer** | | **Viral hepatitis** | | **Influenza and pneumonia** | | **Chronic lower respiratory diseases** | |
| --- | --- | --- | --- | --- | --- | --- | --- | --- | --- | --- | --- | --- | --- | --- | --- | --- | --- | --- |
|  | IRR | 95% CI | IRR | 95% CI | IRR | 95% CI | IRR | 95% CI | IRR | 95% CI | IRR | 95% CI | IRR | 95% CI | IRR | 95% CI | IRR | 95% CI |
| **Model 3: Interaction** |  |  |  |  |  |  |  |  |  |  |  |  |  |  |  |  |  |  |
| CMD x Western | 1.18 | 1.13-1.24 | 1.16 | 1.07-1.25 | 1.12 | 0.99-1.27 | 1.61 | 1.41-1.87 | 1.13 | 1.03-1.24 | 1.22 | 1.11-1.36 | 1.31 | 0.98-1.73 | 1.21 | 1.06-1.37 | 1.19 | 1.08-1.32 |
| CMD x Eastern Europe | 2.04 | 1.94-2.15 | 1.98 | 1.82-2.17 | 2.22 | 1.97-2.50 | 2.09 | 1.78-2.45 | 1.85 | 1.65-2.06 | 1.90 | 1.64-2.19 | 1.08 | 0.89-1.32 | 0.86 | 0.76-0.98 | 1.02 | 0.93-1.13 |
| CMD x Middle East/North Africa | 1.12 | 1.05-1.18 | 0.96 | 0.86-1.08 | 0.92 | 0.83-1.01 | 0.71 | 0.61-0.83 | 0.97 | 0.87-1.09 | 1.28 | 1.08-1.52 | 0.37 | 0.29-0.47 | 1.00 | 0.76-1.32 | 1.15 | 0.87-1.51 |
| CMD x Sub-Saharan Africa | 1.32 | 1.17-1.49 | 1.31 | 1.06-1.61 | 1.27 | 1.06-1.53 | 1.37 | 1.03-1.83 | 1.43 | 1.15-1.78 | 1.75 | 1.22-2.49 | 0.46 | 0.36-0.59 | 0.98 | 0.80-1.20 | 1.01 | 0.85-1.18 |
| CMD x South Asia | 1.28 | 1.19-1.37 | 1.28 | 1.14-1.45 | 1.02 | 0.92-1.13 | 0.99 | 0.80-1.23 | 1.05 | 0.91-1.22 | 1.44 | 1.10-1.88 | 0.59 | 0.45-0.78 | 1.30 | 0.95-1.77 | 1.47 | 1.17-1.85 |

IRR – Incidence risk ratio; 95% CI – 95% confidence interval; CMD – Common mental disorders


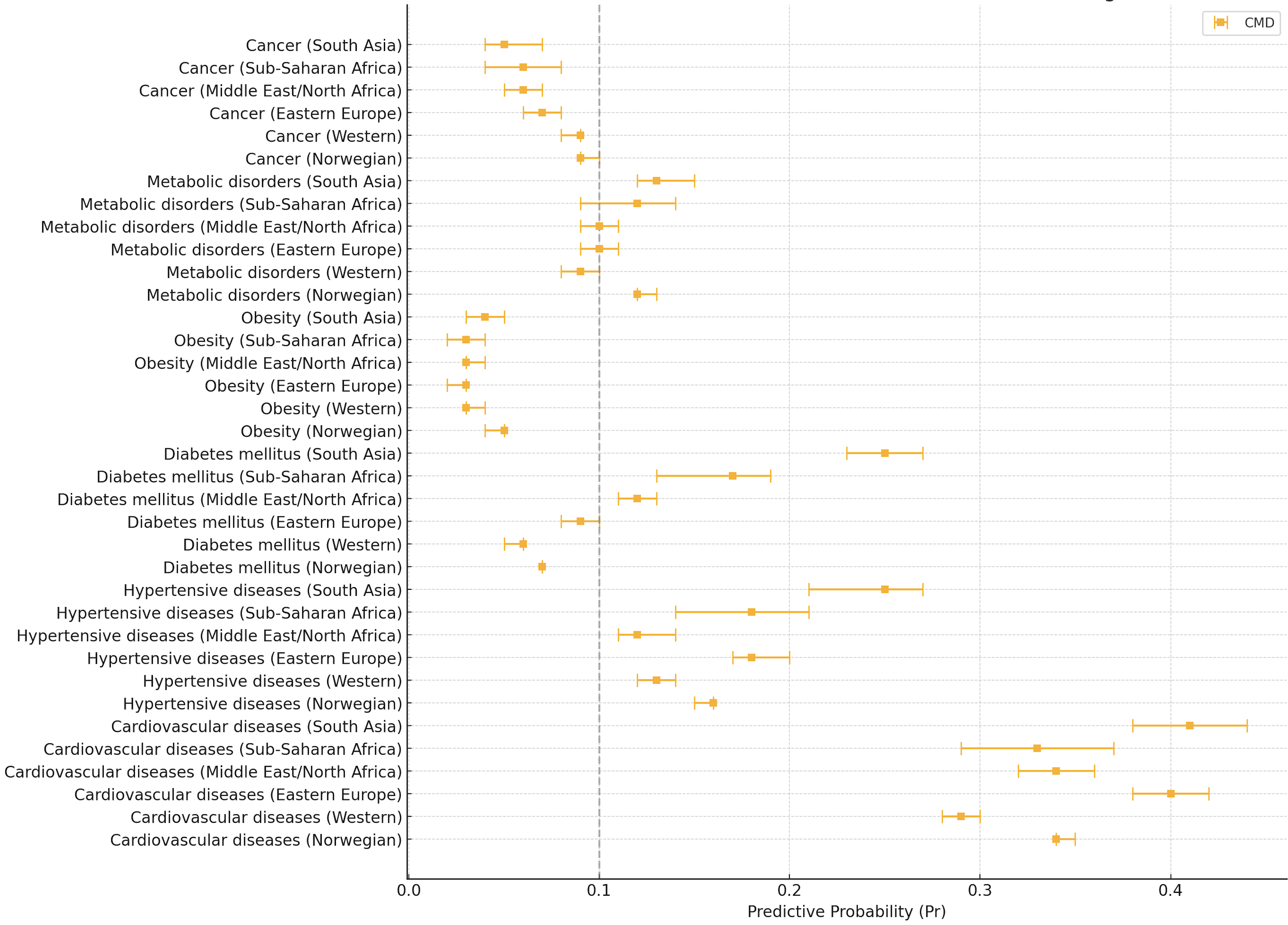


**Supplementary Figure 1**. Predictive probabilities (Pr) with 95% confidence intervals for all diseases across the study groups with common mental disorders (CMD).


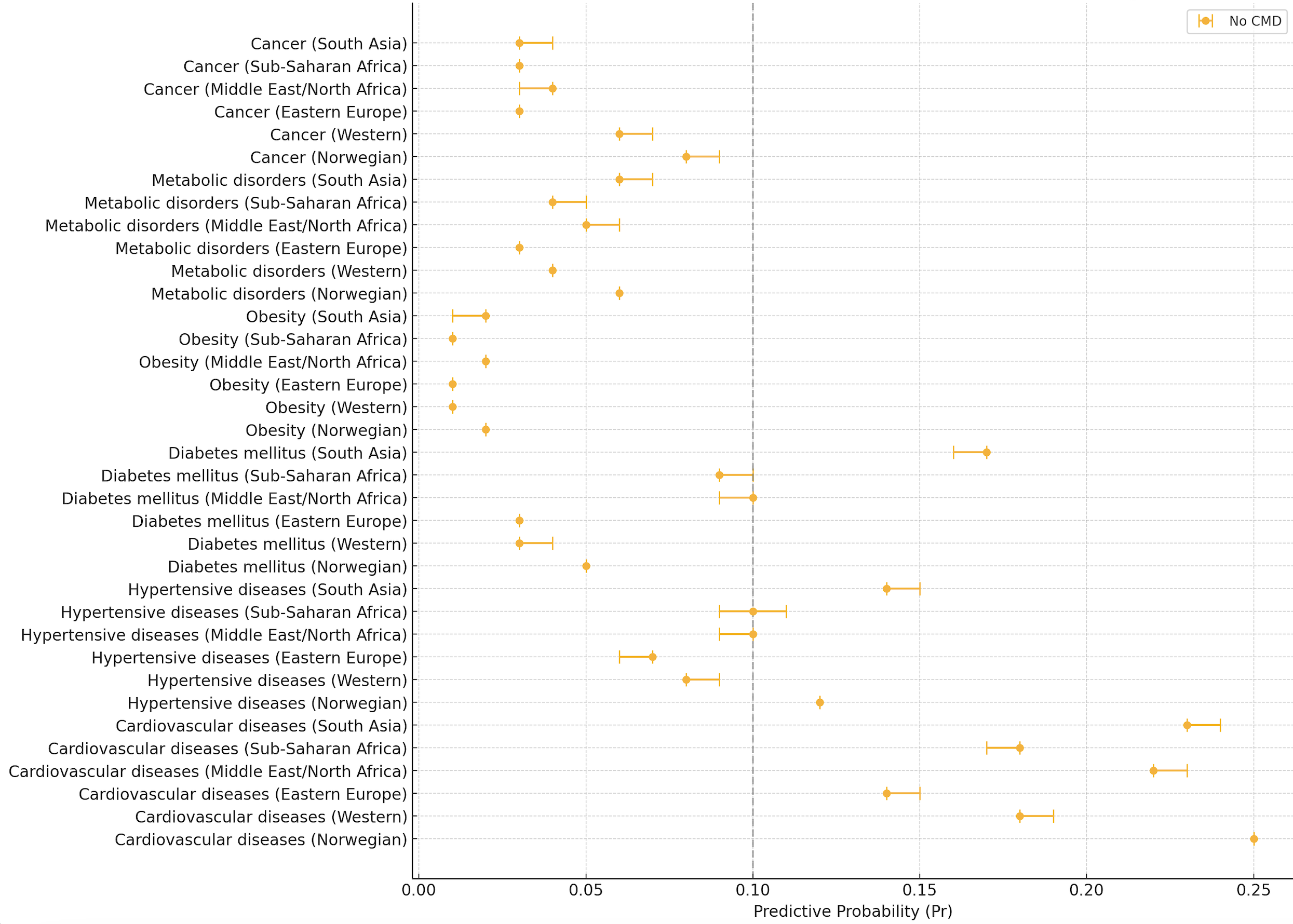


**Supplementary Figure 2**. Predictive probabilities (Pr) with 95% confidence intervals for all diseases across the study groups with no common mental disorders (no CMD).
